# Mitochondrial redox signaling: a key player in aging and disease

**DOI:** 10.18632/aging.204659

**Published:** 2023-04-10

**Authors:** Maria Vitale, Alberto Sanz, Filippo Scialò

**Affiliations:** 1 Department of Molecular Medicine and Medical Biotechnology, University of Naples Federico II, 80131 Naples, Italy; 2 CEINGE, Biotecnologie Avanzate, Naples, 80131, Italy; 3 School of Molecular Biosciences, College of Medical, Veterinary and Life Sciences, University of Glasgow, G12 8QQ, Glasgow, United Kingdom; 4 Department of Translational Medical Sciences, University of Campania Luigi Vanvitelli, 80131 Naples, Italy

**Keywords:** mitochondrial reactive oxygen species, redox signaling, electron transport chain, reverse electron transport, mtROS propagation

The last twenty years have been characterized by findings describing a key role for mitochondrial reactive oxygen species (mtROS) in regulating many physiological processes. These contributions have changed the popular belief that mtROS are just toxic by-products of metabolism. Furthermore, these new findings show that more complex questions have to be asked to fully comprehend the link between changes in mtROS levels and their physiological response. The four essential questions to understand the role of mtROS in health and disease are i) Where are ROS produced? ii) When are ROS produced? iii) How are ROS produced? and iv) How much ROS are produced? These are not trivial questions if we consider that at least eleven sites with the capacity to produce ROS exist in the mitochondrion [1].

ROS generated at different sites regulate different physiological and pathological processes. For example, Complex III (CIII)-derived ROS activates the Hypoxia Inducible Factor 1 alpha, while Complex I (CI)-derived ROS trigger the differentiation of myoblast to myotubes [2]. ROS can be produced at two different sites triggered by different mechanisms, even within the same complex. For example, we and others have demonstrated that electrons are leaked by the flavin site of CI (I_F_) producing harmful superoxide during aging. However, the same complex, under certain conditions produces a ROS signal at the quinone binding site (I_Q_) by a mechanism called Reverse Electron Transport (RET). Interestingly, ROS produced via RET are necessary for stress adaptation, myotube differentiation, macrophage reprogramming and O_2_ sensing [2].

Recently, we have shown that young mitochondria produce ROS in response to specific stimuli such as thermal stress [3]. However, old mitochondria continually produce ROS and don’t respond to stress signals. These findings indicate that mtROS production, like many other processes, is dysregulated during ageing. Furthermore, age-related non-specific ROS cause “noise” that prevents the detection of ROS signals from mitochondria and elsewhere. ROS dysregulation is more detrimental than the simple oxidation of cellular components because oxidized molecules can undergo a recycling process. However suppressing the activation of redox-regulated pathways that trigger transcriptional reprogramming prevents the correct anti-stress response and contributes to increasing age-related mortality [4]. The loss of specific ROS-signalling also explains why antioxidants do not extend animal lifespan [5] and even increase the incidence of certain cancers [6]. Boosting antioxidant levels can reduce oxidative stress, but it cannot restore the essential redox signalling lost during aging.

Therefore, to comprehend the role of mtROS in aging, we need to dissect redox signalling and understand why and how it is lost during aging. The former includes understanding how mtROS signals are propagated to the cytosol and how this process is interrupted during ageing. Several mechanisms have been proposed, including i) the direct oxidation of proteins containing oxidable cysteines by H2O2, ii) the floodgate model, iii) the redox relay model or iv) the crosstalk between mtROS and other ROS generators such as NADPH oxidases [7]. Finally, a non-exhaustive list of targets containing oxidable cysteine residue has being published in recent years [8], shedding light on how redox signalling changes during ageing and showing that mtROS are not simple by-products of metabolism by important cellular messengers essential for a finetuning regulation of both transcriptional and metabolic pathways.
